# The League: A person‐centred approach to the development of social robotics for paediatric anxiety

**DOI:** 10.1111/hex.13981

**Published:** 2024-01-27

**Authors:** Jill A. Dosso, Jaya N. Kailley, Julie M. Robillard

**Affiliations:** ^1^ Department of Medicine, Division of Neurology The University of British Columbia Vancouver British Columbia Canada; ^2^ British Columbia Children & Women's Hospital Vancouver British Columbia Canada

**Keywords:** anxiety, emotion regulation, paediatrics, patient engagement, person‐centred care, social behaviour, social robotics

## Abstract

**Background:**

Social robots are promising tools to improve the quality of life of children and youth living with anxiety and should be developed based on the priorities of end users. However, pathways to include young people in patient‐oriented research, particularly in the overlap between technology and mental health, have been historically limited.

**Objective:**

In this work, we describe engagement with experts with lived experiences of paediatric anxiety in a social robotics research programme. We report the experiences of patient advisors in a co‐creation process and identify considerations for other research groups looking to involve end users in technology development in the field of youth mental health.

**Design:**

We engaged individuals with a lived experience of paediatric anxiety (current, recent past, or from a parent perspective) using three different models over the course of three years. Two initial patient partners were involved during project development, eight were engaged as part of an advisory panel (‘the League’) during study development and data analysis and four contributed as ongoing collaborators in an advisory role. League members completed a preparticipation expectation survey and a postparticipation experience survey.

**Findings:**

Eight individuals from a range of anxiety‐related diagnostic groups participated in the League as patient partners. Members were teenagers (*n* = 3), young adults aged 22–26 years who had connected with a youth mental health service as children within the past eight years (*n* = 3) or parents of children presently living with anxiety (*n* = 2). Preferred methods of communication, expectations and reasons for participating were collected. The League provided specific and actionable feedback on the design of workshops on the topic of social robotics, which was implemented. They reported that their experiences were positive and fairly compensated, but communication and sustained engagement over time were challenges. Issues of ethics and language related to patient‐centred brain health technology research are discussed.

**Conclusions:**

There is an ethical imperative to meaningfully incorporate the voices of youth and young adults with psychiatric conditions in the development of devices intended to support their mental health and quality of life.

**Patient or Public Contribution:**

Six young people and two parents with lived experiences of paediatric anxiety participated in all stages of developing a research programme on social robotics to support paediatric mental health in a community context. They also provided input during the preparation of this manuscript.

## 1 INTRODUCTION

Social robots are a promising emerging technology to deliver interventions in the paediatric mental health space. Defined as devices that can engage in social interaction with a human user,^1^, ^2^ social robots come in a range of form factors, including humanoid, pet‐like, display‐based and completely novel appearances. Social robots for children and young adults in particular are increasingly becoming accessible and affordable,^3^, ^4^ with more than 20 products commercially available as of 2021.^5^ Young people are generally receptive to the idea of robot‐adjunctive mental health therapies.^3^, ^6^, ^7^, ^8^ Social robotics intended to support children with developmental disabilities such as Autism Spectrum Disorder, as well as robotics applications for paediatric distress and anxiety in medical environments have been relatively well researched.^9^, ^10^, ^11^, ^12^, ^13^, ^14^, ^15^, ^16^ The application of robotics to anxiety among children and youth, particularly for ongoing management within a community context, is a newly emerging and promising area.^7^, ^17^, ^18^

Researchers and scientific organizations, in Canada and globally, increasingly recognize the ethical imperative to collaborate with persons with lived experience during all stages of research concerning them.^19^, ^20^, ^21^, ^22^, ^23^, ^24^, ^25^, ^26^ This can include patients and persons with lived experiences playing a role in setting research priorities, selecting research designs and interpreting and disseminating findings.^27^ Such end‐user engagement in research helps ensure that studies are appropriate and relevant for patient communities.^28^ However, pathways to involve patients and persons with lived experience in healthcare technology research from the early stages of innovation are limited,^29^ and this challenge is even greater when it comes to children and youth. Researchers are encouraged to engage young people directly, rather than relying on adult counterparts as proxies, as young people have their own unique perspectives to bring to the table.^30^, ^31^ For example, feedback from children on the quality of an interaction can contrast with adult reports of the same event.^32^, ^33^ It is therefore urgent to understand what is most important to both children and families when it comes to social robotics and children's mental health to ensure that these newly popular technologies are deployed in an ethical, evidence‐based and patient‐centred manner. However, at present, social robotics are often developed and implemented without substantial end‐user feedback.^34^, ^35^

The present work falls into the categories of digital technology and co‐creation in the ‘mosaic of neuroethics’.^36^ Informed by a neuroethics framework, it is intended to evaluate, on an ongoing basis, whether the benefits of companion robots for brain health outweigh the risks and respond to this balance by informing future research, implementation and policy in this area. We also prioritize reflexivity as a value—a process where researchers describe their position in the work with respect to the topic and other individuals involved. Reflexivity provides context for the study and allows for greater understanding of findings.^37^, ^38^, ^39^

Here, we provide a model for a path towards patient engagement in robotic development for mental health support for children and youth. In the present work, we describe three stages of patient partnership with youth, young adults and parents with lived experiences of paediatric anxiety in service of research on social robotics for child mental health support. The techniques described here may be informative for others working towards the evidence‐based deployment of new technologies intended to support children and youth with psychiatric conditions.

## 2 MATERIALS AND METHODS

### 2.1 Engagement context

The scientific team included three university‐based Canadian academic women (a principal investigator, a postdoctoral fellow and an undergraduate student) with experience working with children and youth in research settings to investigate topics around health and technology using qualitative and quantitative methods. The League was formed as part of a larger body of work, the SIDEKICK project, concerned with social robotics and paediatric anxiety, some of which has been reported elsewhere.^40^

### 2.2 Models of patient engagement

**Figure 1:**
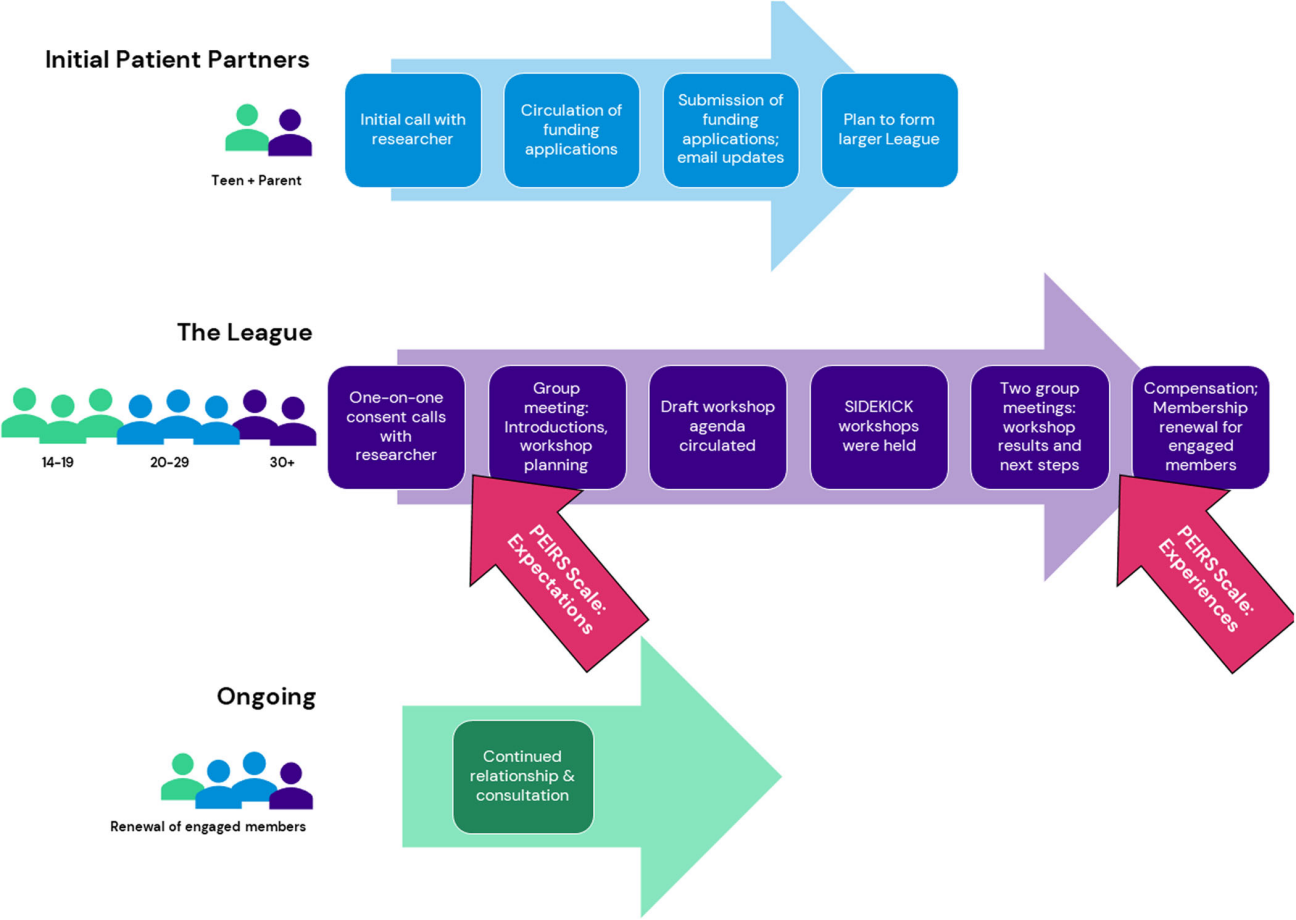
Key patient engagement events and participants.

Ethics approval was obtained for the League and subsequent group activities. Ethics approval was not sought for the initial patient partner phase for reasons covered in Section 4. Written informed consent was obtained from all adult participants, and minors provided assent combined with parent consent. As part of a larger body of work on social robotics, individuals with present and past lived experiences of paediatric anxiety were engaged using three different models (Figure 1).

#### 2.2.1 Initial patient partners

We initially worked with Foundry BC, an organization that provides youth mental health support, to connect with two individuals with a lived experience of anxiety: a teenager and their parent. After an initial discussion with the research team and an orientation to the topic of social robots and their potential applications for paediatric mental health, the family was named patient partners in funding applications to support the subsequent work, and we corresponded with them via video call and email. This arrangement was implemented from September 2020 to May 2021.

#### 2.2.2 Panel of advisors

Once the research proposal was funded, we convened a full panel of advisors, which we named the Lived Experience Expert Group (LEEG, ‘the League’), to advise on all aspects of the SIDEKICK project, including recruitment, workshop design and knowledge translation. Foundry BC circulated information about this opportunity to families connected to the organization. Populations to be recruited were (1) children (7–18) with self‐disclosed lived experience of paediatric anxiety, (2) family members (all ages) of individuals (7+) with lived experience of paediatric anxiety and (3) young adults with lived experience of anxiety during their own recent childhood/youth. All members had to be able to read, write and speak in English and possess sufficient computer skills to participate in online video calls and correspond via email.

Individuals who expressed interest were sent material explaining the intended format, goals and expectations for the League. Those who remained interested then filled out a pre‐engagement survey in May 2021 to provide information about their expectations, concerns and preferred modes of communication (Appendix S1), followed by a one‐on‐one phone or video call with a researcher. During these calls, the researcher worked to build rapport, introduced the engagement topics of interest, provided an orientation to social robotics as a research area, explained the intention to create a panel of lived experience experts and answered any questions that arose during the call. Responses from the pre‐engagement survey scaffolded this discussion. Finally, the researcher summarized the consent/assent form, and individuals were able to reflect on the call before submitting their consent/assent online in their own time.

After opting to join the League, individuals were invited to participate in an initial meeting over Zoom in August 2021 to finalize the workshop procedures for the SIDEKICK project. The agenda for this meeting was developed in consultation with a clinical psychologist specializing in paediatric anxiety. The goal of the meeting was to allow members to get to know each other, introduce the field of social robot research and demonstrate specific examples of what social robots look like and can do and gain insight into how to conduct this research in a way that would be well suited to families living with paediatric anxiety. Participants also completed an anonymous but shared poll about their experiences living with anxiety as a rapport‐building exercise and to inform the discussion. Based on the feedback received during the Zoom meeting, the researchers developed a SIDEKICK workshop agenda and circulated it via email to all the League members for their input.

SIDEKICK workshops (*n* = 56 individuals with lived experiences of paediatric anxiety) were conducted in Fall 2021 and Winter 2022 and results are reported elsewhere.^40^ In May 2022, after the SIDEKICK workshops were completed, a Zoom meeting was held with the League to share workshop findings and discuss analysis and next steps. In June 2022, a survey with select questions from the Patient Engagement in Research Scale (PEIRS)^41^ was circulated to all members as a postengagement measure of experiences. Members were provided a $400 honorarium for their participation over the year.

#### 2.2.3 Retention of engaged members

The four League members who displayed sustained interest through the full term were invited to renew their membership. We continue to engage them in a more format‐flexible dialogue via email and one‐on‐one calls around ongoing research and engagement projects in the lab and the creation of the present document.

## 3 FINDINGS

### 3.1 Membership

Initial patient partners were a parent and a teenage child. They later joined the larger League, which included six additional participants: two teenagers living with anxiety, three individuals in their twenties with a recent experience of anxiety during their childhood and a parent of a child with anxiety. Two individuals self‐disclosed a diagnosis of Autism Spectrum Disorder. Three individuals disclosed that their anxiety was related to physical health diagnoses or procedures performed in their childhood. Three individuals shared that they had volunteered in a similar advisory role in the past, and many individuals were working or studying in a health‐ or mental health‐adjacent field. Four particularly engaged League members were renewed for the third stage of partnership, including the original patient partner family.

### 3.2 Pre‐engagement survey findings

From the League pre‐engagement survey, we learned that members were interested in options to contribute to the engagement process in writing, or in exchanges without video. Participants were generally hopeful that they would be able to make meaningful contributions to the project, and they felt that participating in the League would be a positive experience. Almost all members expressed an interest in getting to know the other members, and this led to the decision to hold an initial group kick‐off Zoom meeting rather than engage the group asynchronously. In terms of reasons for participating, members shared that they wanted to help others, gain new perspectives on anxiety, learn to manage their anxiety, support research and meet others with similar experiences.

### 3.3 Advising the SIDEKICK workshops

Seven of eight League members were able to attend the kick‐off Zoom meeting. Discussion was augmented through online poll, chat and anonymous response tools, and these text responses were saved. While the verbal discussion was not recorded, one researcher acted as a note‐taker. The nonattending League member contributed via email.

The first topic of discussion was about difficulties resulting from living with anxiety, posed as an anonymous question. This question served to identify shared experiences within the group and to spark ideas about applications of social robotics for anxiety. Themes included difficulties communicating with others about experiences and feeling misunderstood, finding everyday life difficult and a desire to isolate oneself. Next, the discussion covered the mechanics of how the SIDEKICK workshops should be constructed (Table 1). After recommendations from the kick‐off meeting had been incorporated in a workshop agenda, this was circulated to League members for further comment (Table 2).

**Table 1:** League recommendations for workshop format.

Recommendation	Outcome
Small (4–6 people), but not one on one	Implemented
Multiple response options: chat, multiple choice questions, polls, anonymous responding	Implemented
Not too loud; not too noisy	Implemented
Allow participants to bring a support person	Partially implemented^a^
Recruit groups of participants with pre‐existing relationships to each other	Not implemented^b^
Keep age groups narrow within workshops	Implemented^c^

^a^ Children 7–13 brought family members; youth 14–18 did not.

^b^ Ethically challenging.

^c^ Workshops had a maximum of 4 years' difference between participants, with the exception of siblings.

**Table 2:** League comments on draft workshop agenda.

Recommendation type	Quote
Language and terminology	‘I found the language to be a bit more difficult if you are working with a younger group, particularly the 7–13 year olds as you will have some 7–8 year olds who may not be completely familiar with how to describe their anxiety or emotions. Elaborating on those questions or providing examples may be useful. Like when asking “what is one difficult thing about living with anxiety?” maybe providing an example like “it's hard to make friends” could help spark the discussion’.
Activity ideas	‘Have them find something in their environment that they turn to when they are anxious, or something that speaks to them when they get anxious, and maybe that will provide some insight to what might be helpful?’.
Recruitment avenues	‘it may be worthwhile reaching out to nurse clinicians in the outpatient clinics (ex. GI, rheumatology, endocrinology, healthy minds centre, etc.)’
Response mechanics	‘I appreciate that you made it so the more personal things are anonymous’.

Only four of the eight League members were able to participate in meetings to discuss the SIDEKICK results. Members reported that the workshop discussions mirrored their own preferences and experiences. They expressed surprise that younger workshop members were easily able to imagine how a robot would fit into their life and that their views on privacy were so sophisticated. They echoed specific SIDEKICK workshop findings: the utility of a robot for companionship, reminders and alarms and a preference for a social robot to have a warm, pet‐like form factor.

### 3.4 Evaluating League experiences

Three League members (of eight who were invited) completed the anonymous postengagement survey with select questions from the PEIRS.^41^ All agreed with statements including ‘I understood the objectives of the project’, ‘Participating in the League was a positive experience’ and ‘I was offered fair compensation for my participation in the League’. Two of three agreed with the statements ‘The time commitment for participating in the League was reasonable’, ‘Throughout this project, communication with the research team was clear’ and ‘I felt that my thoughts and opinions were valued in the League’. They also provided written feedback (Table 3).

**Table 3:** Written feedback from League members.

Type of feedback	Responses
Benefits of participating	It was a good insight into how mature and aware the younger patients are when it comes to explaining their needs to deal with anxiety.
	Yes, I benefited from being in the League by being able to voice my opinions and collaborate with the team on ways to help the workshops and research.
Favourite part	Definitely the part where we got to pick and choose the robots and how they interact with participants.
	I very much enjoyed the brainstorming and reviewing the content from the workshops.
Least favourite part	It would be great to sit into a workshop session just to see everything live!
	I did not have a least favourite part of being in the League.
Concerns	Just the possibility that there may be a biased filter as a researcher as to how to frame the scope/approaches to anxiety because the kids may be the better teachers and are more capable than what we take them for!
	No concerns.
Suggestions for improvement	Take a step back and address anxiety as a holistic approach so that the benefits can expand beyond kids, but also applicable when navigating life when the anxiety levels become less pervasive.

## 4 DISCUSSION

This work presents an approach to developing paediatric health technologies that centre the perspectives of youth and young adults with anxiety through three models of patient engagement: a single patient partner family engaged early in the funding application stage, an advisory panel of eight individuals who informed a specific research project and a set of four engaged patient partners participating in a semistructured relationship with the research team to inform ongoing work. Findings suggest that this type of collaboration can offer diverse, valuable and actionable insights to inform research practices. Here, we reflect on the language choices that are relevant to this type of work, the relationship between patient engagement and formal ethics bodies, the strengths and limitations of the present approaches and recommendations for research community members interested in similar activities.

### 4.1 Language

We have used both ‘patient’ and ‘person with lived experience’ in the present document. All of our patient partners reported that they or their child had a ‘lived experience of anxiety’ as part of the recruitment process, and initial advertisements for the project were circulated by a youth mental health support resource. We did not require a clinical anxiety‐related diagnosis to participate, and we did not formally inquire about this information. Most participants chose to spontaneously self‐disclose a diagnosis to the research team and to other League members. While several self‐disclosed past experiences within the medical system, these were more likely to cause or aggravate anxiety, rather than result from it. One model predicts that an individual's relationship to the term ‘patient’ is related to their past experiences of medical treatment.^42^ The research team is centred within the ‘Patient Experience’ unit in a Children's Hospital, receives funding from sources that use the ‘patient‐oriented research’ language, and has presented this work in outlets specifically focused on ‘patient partner’‐related activities. As such, we incorporate this language to maintain our link to these professional bodies. However, others have reported that the word ‘patient’ can pose a barrier to engagement with lived experience experts; by emphasizing illness or deficit rather than expertise, the term can be seen as undervaluing and disrespecting these individual's contributions to research and to the engagement process.^43^ We spoke with three of the ongoing League members about this topic. Two reported that they preferred the term ‘lived experience experts’ as it included individuals without a formal diagnosis and better reflected day‐to‐day life outside of a hospital context, while one reported that she was ‘okay with both terms’.

### 4.2 Role of Research Ethics Board

In addition to questions of language, the topic of how best to involve lived experience partners in work such as the project described above—how to recognize their expertise and contributions while respecting potential vulnerability related to mental health history—has implications for choices around institutional ethics approval.^44^, ^45^ If patient collaborators are considered as equivalent to professional peers, then obtaining approval from a Behavioural Research Ethics Board, Institutional Review Board or similar body to engage with them as human subjects is inappropriate. However, they may also be considered vulnerable to harms as a result of their engagement in the research process, especially if they are young or being asked to discuss their physical or mental health history. The Canadian Institutes of Health Research states that partnering with patients at the planning and designing stages of research does not require ethics approval.^46^ Therefore, we applied for and received ethical approval for the present work at the point when we formed the League advisory panel (which involved discussing summarized study results) but not for the work with the two initial patient partners.

### 4.3 Strengths and limitations of the present approach

The present approach featured three different formats of consultation with lived experience experts. The adoption of multiple engagement formats within a line of work is often desirable within patient‐oriented research.^47^ However, involvement of patient partners at the funding and research question development stages, as we did, is on the rise but not yet common.^47^ In our case, the two initial patient partners were instrumental in defining research questions and obtaining the financial support needed to run the rest of the project. Viewing robotics research through the lens of Neuroethics, there is an imperative to ensure that the priorities of robotics researchers are aligned with the values of future end users of the products. Integrating the perspectives of persons with lived experiences of anxiety throughout the research process, from conception to analysis, fits within this framework. Nevertheless, this approach includes challenges. First, existing laboratory funds were needed to compensate these individuals for their contributions to an initially unfunded project. Second, while asking partners to join the project early on before many specifics had been ironed out allowed them to have a larger role in determining the direction of the work, it also required them to take a ‘leap of faith’ in joining a line of research that was not yet fully defined and subject to uncertainty around factors such as timeline and funding.

The present work relied on consulting with a relatively small number of patient partners in total. While we did not formally collect gender, income or race/ethnicity information, it is our impression that the membership was somewhat diverse on these metrics but not fully representative of the population being sampled. We chose not to collect detailed demographic information from the patient partners to honour their role as collaborators rather than research subjects. This aligns with work finding that some engagement partners find this type of demographic data collection to be unnecessary, intrusive, or inappropriate, given their role.^48^ However, this choice did limit our ability to evaluate the representativeness of the group to our population of interest. Furthermore, our reliance on a nonrandom set of volunteers does introduce the possibility of bias, but is a common practice in the field of patient engagement.^28^ Other models of patient engagement offer different advantages and disadvantages; consulting with a large number of patients at a single time point is logistically difficult but allows for more perspectives to be heard.^49^ On the other hand, extended discussions with a small number of invested persons through deliberative consultation allow for more in‐depth insight into a single issue.^50^, ^51^

While our ultimate research questions were around developing social robots for a paediatric population (i.e., 18 years and younger), and the workshops that we developed through this patient advisory process ultimately collected data from children and adolescents, many of our patient partners were adults. In particular, in addition to our three paediatric members, we included three individuals in their 20s (ages 22, 22 and 26). These young adult advisors were all recruited through their connection with a youth mental health organization that had provided services to them while they were 18 years or younger, and all self‐reported lived experiences with anxiety during their childhood. Due to the challenges that we experienced in recruiting children and teenagers for long‐term participation in our advisory group, we felt that these ‘near peers’ could still provide valuable insights for our team. We also included two adult parents of children currently living with anxiety as patient partners. Parents are commonly recognized as valuable experts on their children's health^52^, ^53^ and they provided key insights about factors affecting families' ability to participate in the research being planned.

The format of the League group—an initial meeting with introductions and icebreakers, an informal focus group format, ongoing email communication and a 1‐year renewable term—is in line with other health‐related youth advisory groups in our research context.^54^ Like others, we shifted to an online format as a COVID‐19 adaptation.^54^ Because of the format, we were able to recruit a more geographically diverse sample than would otherwise have been possible, which has been recognized as a past challenge in this type of patient engagement.^54^ League members were motivated to participate for a number of reasons, in line with experiences reported by other research groups: wanting to help others, to connect with others who share similar experiences, and to gain professional experience.^55^, ^56^, ^57^

Maintaining engagement with members of the League was a challenge, an experience also shared by other research groups.^54^ Meetings were not attended by all members, and individuals were unevenly able to engage by email. We also encountered logistical challenges consistent with others.^26^ This included staff turnover on the part of the academic team (e.g., students graduating, parental leave), posing a challenge for long‐term relationship building with patient partners. Keeping a focus on childhood experiences of anxiety was a priority for us, and this required a nuanced approach.^58^ The tendency of adults to speak on behalf of their children was both a benefit and a challenge—this allowed younger members' contributions to be relayed even when they did not feel comfortable speaking aloud but may have also created an atmosphere where adult perspectives dominated. Another limitation of our work with the League was the limited feedback that we received about their experiences. Several chose not to complete the final survey, so their perspectives are absent. While the final survey was anonymous, the small size of the group means that individuals wanting to provide less positive feedback may have been concerned about revealing their identity, which limits our ability to interpret the data that we did collect.

The present work was successful in that patient partners provided feedback that was actionable and resulted in real changes to the resulting SIDEKICK workshops.^40^ Participants also agreed that their involvement was a positive experience, that they benefited by being able to voice their opinions and that they enjoyed contributing. The perspectives gathered through engaging the League were instrumental in informing the format and content of the workshops. As a result, the research team felt confident in the quality of the data generated by this approach.

### 4.4 Comparison to an older adult League

Our research group also works with a League (LEEG) comprised of older adults and current and former care partners of persons living with dementia.^2^, ^59^ To briefly compare our experiences with the two groups: both groups wanted to meet other members with similar life experiences, and were motivated to help others, to participate in research, and learn about new technologies. Both groups have a variety of levels of participation across members. The older adult group, being mostly retired, prefer to meet during the workday, while the younger group was mostly available during evenings and weekends. The older adult group seemed to prefer a higher frequency of communication overall compared to the younger group, who were balancing school, work and family commitments. The younger group enthusiastically embraced and requested a variety of response methods and online discussion tools (e.g., polling software, interactive Q&As), whereas the older group preferred more straightforward written or spoken communication. We accommodated to these different preferences and expectations based on initial expectations surveys given to both groups as well as real‐time feedback.

## 5 CONCLUSION

We engaged with persons with present and past lived experiences of paediatric anxiety to co‐create a social robotics research programme. Resulting patient engagement practices were strengthened, and partner voices were heard. Facilitators of success included providing multiple flexible pathways for partners to contribute (e.g., video, text, online interactive activities), consulting with a variety of age groups and incorporating partner feedback early in the engagement cycle. Best practices around language and ethics in this space are still evolving. Development of new technologies for paediatric populations with psychiatric conditions will benefit from a person‐centred approach at all stages of research.

## AUTHOR CONTRIBUTIONS

**Jill A. Dosso**: Conceptualization; data curation; formal analysis; funding acquisition; investigation; methodology; project administration; visualization; writing—original draft; writing—review & editing. **Jaya N. Kailley**: Data curation; investigation; formal analysis; writing—original draft; project administration. **Julie M. Robillard**: Conceptualization; methodology; data curation; investigation; supervision; funding acquisition; project administration; writing—review and editing.

## CONFLICT OF INTEREST STATEMENT

The authors declare no conflict of interest.

## ETHICS STATEMENT

The University of British Columbia Behavioural Research Ethics Board provided approval for the League and subsequent research activities (ethics approval was not sought for the initial patient partner phase for reasons covered in Section 4 of this document). Written informed consent was obtained from all adult participants, and minors provided assent combined with parent consent.

## Supporting information

Supporting information.

## Data Availability

The data that support the findings of this study are available on request from the corresponding author. The data are not publicly available due to privacy or ethical restrictions.
