# Supplementary material for: The League: A person‐centred approach to the development of social robotics for paediatric anxiety
Source: Health Expect. 2024 Jan 27;27(1):e13981. doi: 10.1111/hex.13981 (PMC10821745; doi:10.1111/hex.13981)
Supplement: Supplementary file 1 — Supporting information. [file HEX-27-e13981-s001.docx]

# Appendix 1

League Pre-Engagement Survey

*Thank you for taking the time to fill in this survey! We would love to get a sense of your hopes, expectations, and possible concerns about the League. There are no right or wrong answers. You may skip any question you don't feel comfortable answering.*

*Your responses will only be viewed by the research team, and we'll schedule a phone call to chat about your potential participation in the League soon.*

*If you have any questions or concerns in the meantime, feel free to reach out to the member of the research team you have already been talking with by email.*

1. The time commitments for participating in the League seem reasonable to me.

- Strongly agree
- Somewhat agree
- Neither agree nor disagree
- Somewhat disagree
- Strongly disagree

1. My preferred mode of communication for League activities would be (choose all that apply):

- Email
- One on one calls (audio only) with a member of the research team
- One on one video calls with a member of the research team
- Conference calls (audio only) with the research team and other League members
- Video calls with the research team and other League members
- In-person meetings with the research team and other League members

1. Do you have any additional comments or concerns about modes of communication?

[free response]

1. I think I will feel comfortable expressing my thoughts and opinions in the League.

- Strongly agree
- Somewhat agree
- Neither agree nor disagree
- Somewhat disagree
- Strongly disagree

1. What can we do to make you more comfortable?

[free response]

1. I feel that my thoughts and opinions will be valued in the League.

- Strongly agree
- Somewhat agree
- Neither agree nor disagree
- Somewhat disagree
- Strongly disagree

1. I expect that I will be able to contribute to the League in a meaningful way.

- Strongly agree
- Somewhat agree
- Neither agree nor disagree
- Somewhat disagree
- Strongly disagree

1. I expect that participating in the League will be a positive experience.

- Strongly agree
- Somewhat agree
- Neither agree nor disagree
- Somewhat disagree
- Strongly disagree

1. In what ways do you hope to benefit from being in the League?

[free response]

1. What do you most look forward to about participating in the League?

[free response]

1. What are your concerns about participating in the League?

[free response]

1. Would you be interested in getting to know the other members in the League?

- Yes
- Maybe
- No
